# A Novel Dry Model for Practicable Sphincterotomy and Precut Needle Knife Sphincterotomy

**DOI:** 10.1155/2014/908693

**Published:** 2014-09-10

**Authors:** Akio Katanuma, Takao Itoi, Junko Umeda, Ryosuke Tonozuka, Shuntaro Mukai, Kei Yane, Toshifumi Kin, Kazuaki Matsumoto, Tomoaki Matsumori, Katsushige Gon, Ryo Takaki, Akiko Tomonari

**Affiliations:** ^1^Center for Gastroenterology, Teine-Keijinkai Hospital, 1-40-1-12 Maeda Teine-ku, Sapporo 006-8555, Japan; ^2^Department of Gastroenterology and Hepatology, Tokyo Medical University, Tokyo 160-0023, Japan

## Abstract

*Aim*. We aimed to develop a simulation dry model for endoscopic sphincterotomy (ES) and needle knife precut sphincterotomy (NKP) and to evaluate its usefulness as a training simulator. *Materials and Methods*. An endoscopic retrograde cholangiopancreatography trainer was used as a duodenum, bile duct, and papilla simulator. A simulated papilla was created with a piece of rolled uncured ham, and ES and NKP were performed. Hands-on training was carried out using this model, and success and failure of the procedures were evaluated. A questionnaire survey was conducted among the participants to assess the performance and usefulness of the dry model for ES and NKP training. *Results*. Twenty-two endoscopists participated in the hands-on training using this dry model. ES was successful in 33 out of 34 attempts (97%) whereas NKP was successful in all 7 attempts (100%). Based on the results of the questionnaire survey, the median score for realism was 7 (range: 2–9) for ES and 8 for NKP on a scale of 1 to 10. *Conclusions*. The dry model using an uncured ham provides a condition closely similar to actual clinical practice and is useful as a training model for ES and NKP.

## 1. Introduction

Endoscopic sphincterotomy (ES) is one of the most frequently performed procedures by endoscopic retrograde cholangiopancreatography (ERCP) endoscopists. ES is indicated for many diseases, such as lithotomy of bile duct stones and for bile duct stenting [1, 2]. Needle knife precut sphincterotomy (NKP) is performed as an alternative method when cannulation of the bile duct by conventional methods is difficult [3, 4]. Although ES is frequently carried out, the rate of complications (e.g., bleeding and perforation) approximately ranges from 3% to 10% [5–10]. NKP is a more difficult and challenging procedure, especially for beginners.

To acquire and master the necessary skills for the ES and NKP techniques, accumulating experience in actual clinical practice in many institutes is needed. Admittedly, the lack of sufficient training and experience may increase procedural failures and complications. To resolve such problems, various simulators have been developed, including computer simulators [11], ex vivo porcine organs with the Erlangen Endo-Trainer [12], and an anesthetized pig model [13]. Although computer simulators are applicable to various endoscopic procedures, the models are expensive and difficult to apply for routine use. Furthermore, computer simulators provide little realism of cutting. Ex vivo porcine organs with the Erlangen Endo-Trainer are reported to be useful, but the Erlangen Endo-Trainer requires fluoroscopy for visualizing procedures within the biliary system. Although models using anesthetized pigs provide the realism of cutting, equipment for animal experiments is necessary as well as various preparations (e.g., anesthesia induction). Furthermore, the location of the papilla is anatomically different between humans and pigs. Each training model has its own features and no optimal model has been developed to date.

We developed a dry model for ES and NKP training. The model was relatively cheap and easy to prepare and use. Hands-on training was conducted to evaluate the usefulness of this dry model for ES and NKP simulation.

## 2. Materials and Methods

### 2.1. Preparations of the Dry Model

**Figure 1:**
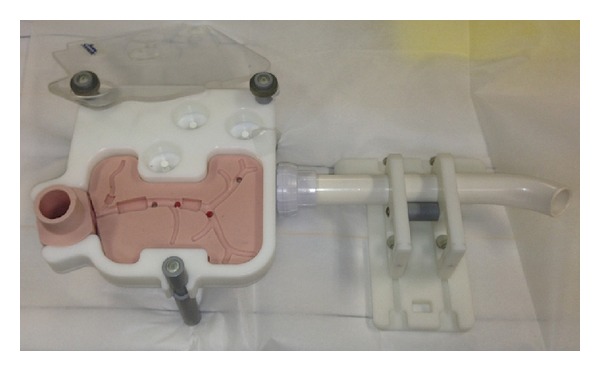
Endoscopic retrograde cholangiopancreatography trainer (the Chamberlain Group, LLC, Great Barrington, MA, USA).

**Figure 2:**
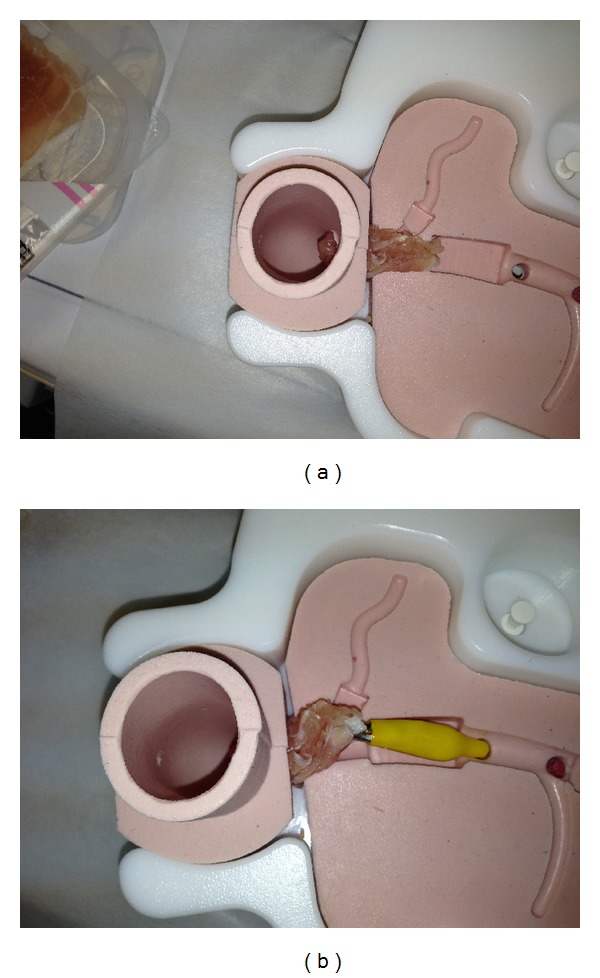
Creation of the simulated papilla. (a) A piece of rolled uncured ham was inserted into the opening with the tip protruding. (b) For the transmission of current, the model was earthed via a conventional cable connected to the simulated papilla.

An ERCP trainer (the Chamberlain Group, LLC, Great Barrington, MA, USA; Figure 1) was used as a duodenum, bile duct, and papilla simulator. This simulator allows the insertion of a duodenoscope and was developed for cannulation training to the bile duct. The papillary part is an opening connected to the bile duct. A piece of rolled uncured ham was inserted into the opening with the tip protruding 1 cm to create a simulated papilla. For the transmission of current, the model was earthed via a conventional cable connected to the simulated papilla (Figure 2).

### 2.2. ES and NKP Procedures

**Figure 3:**
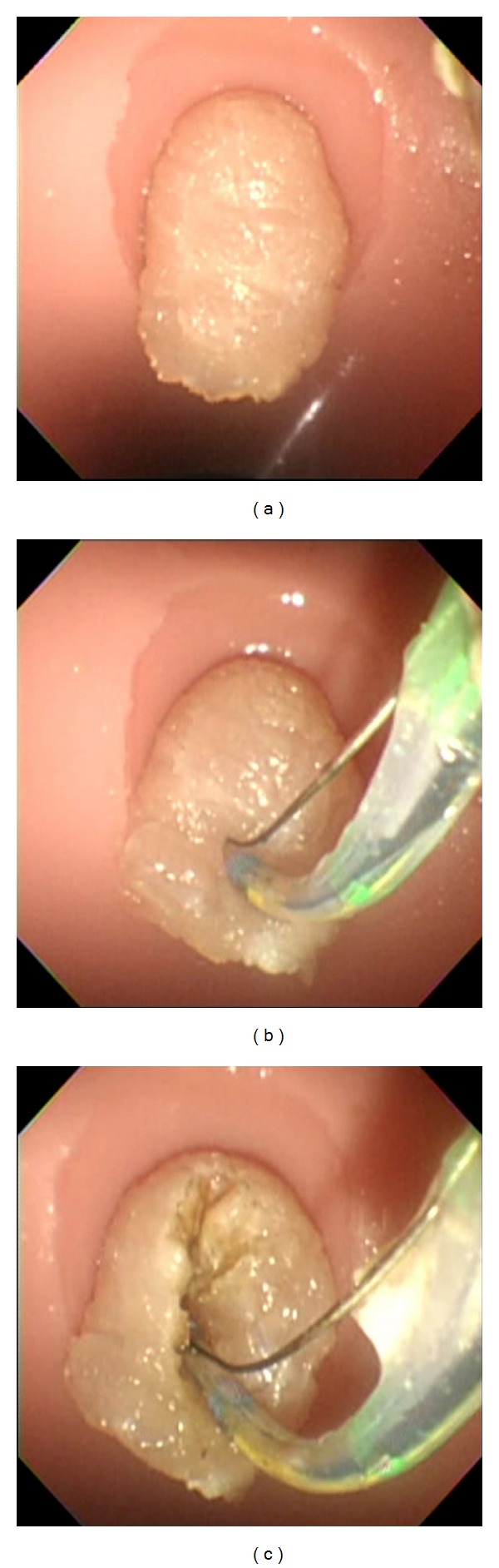
(a) Endoscopic observation of the papilla. (b) Insertion of the Autotome into the simulated papilla. (c) Performance of ES while the cutting direction was controlled by manipulating the endoscope and elevator function.

**Figure 4:**
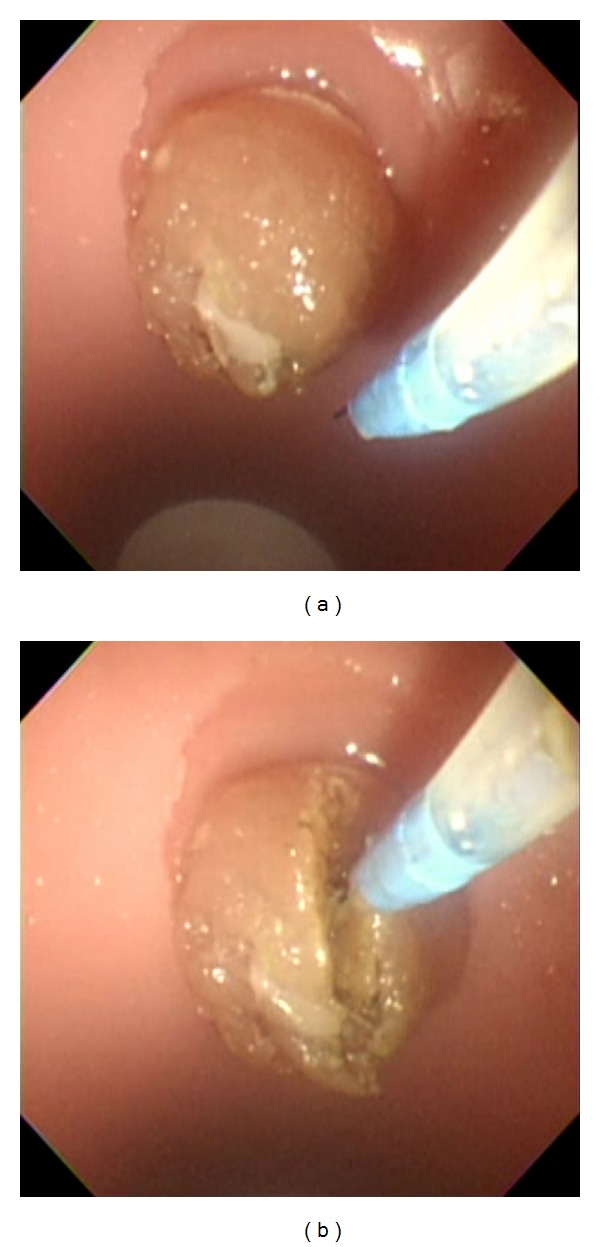
(a) Performance of NKP using an RX knife (Boston Scientific). (b) Performance of NKP while the cutting direction was controlled by the elevator function.

**Figure 5:**
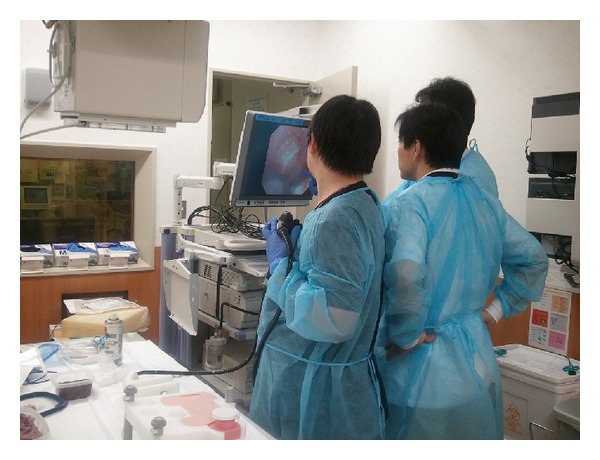
Hands-on training using the dry model. A trainee performed ES with the assistance of an experienced ERCP endoscopist.

ES was performed using JF-260V (Olympus Medical Systems, Tokyo, Japan) as an endoscope and Autotome (Boston Scientific, Fremont, CA, USA) as a papillotome. When the endoscope was advanced to the duodenum of the simulator, the papilla was observable. From this site, the papillotome was inserted into the bile duct under the guidance of a guidewire. The simulator was equipped with a simulated bile duct, allowing the introduction of the guidewire. Because an uncured ham was used, there was no actual bile duct orifice. Although cannulation was possible from any part of the simulated papilla, only cannulation from the appropriate site, that is, around the center of the simulated papilla, allowed the guidewire to be guided into the simulated bile duct. Electrosurgical generators (ICC 200; ERBE Elektromedizin, GmbH) were used to perform ES and NKP in which the effect was 3 and the Endocut was set at 120 W. After cannulation, the papillotome was positioned at the appropriate site of the simulated papilla similar to the usual ES procedure. ES was performed while the cutting direction was controlled by manipulating the endoscope and elevator function (Figure 3). After completing each procedure, pieces of uncured ham were exchanged in a short period of time of less than 3 minutes. NKP was performed using an RX knife (Boston Scientific) and the electrosurgical generators were set up similarly as in the ES procedure (Figure 4). Two instructors (Akio Katanuma and Takao Itoi) were always present during each procedure. The instructors mainly gave advice to the beginners on how to manipulate the scope, sphincterotome, and needle knife sphincterotome (Figure 5).

### 2.3. Evaluations

Hands-on training was conducted using the dry model at 2 referral centers in Japan (Center for Gastroenterology, Teine-Keijinkai Hospital, and the Department of Gastroenterology and Hepatology, Tokyo Medical University) with 22 endoscopists. After each endoscopist performed the procedures several times, a questionnaire survey was conducted to collect feedback on endoscope manipulation and ES and NKP performances on the dry model compared with those in actual clinical practice. The contents of this questionnaire survey (Table 1) aimed to investigate how many times successful cutting was achieved when ES was performed on this dry model. Successful ES was defined as completed cutting to the upper site of the simulated papilla to the right direction such as towards the 11 to 12 o'clock direction. Successful NKP was also defined similarly. Two instructors (Akio Katanuma and Takao Itoi) assessed whether successful cutting was achieved or not. Furthermore, the following items were assessed on a scale of 1 to 10 based on the subjective views of the endoscopists: how the manipulation of the endoscope on this dry model was graded, whether the dry model provided the realism of cutting as in the actual ES and NKP procedures in clinical practice, whether the participants considered the model to be helpful for improving their skills in performing the ES and NKP techniques, and whether they considered the dry model to be helpful for improving beginners' skills in performing the ES and NKP techniques. Each of these items was analyzed to determine whether there were differences in scores according to the number of years of ES experience.

## 3. Statistical Analysis

All statistical analyses were performed using EZR (Saitama Medical Center, Jichi Medical University), which is a graphical user interface for R (The R Foundation for Statistical Computing, Vienna, Austria, version 2.15.3). More precisely, it is a modified version of R commander (version 1.9-5) designed to add statistical functions frequently used in biostatistics. The Mann-Whitney *U* test was used to compare categorical variables based on the ES experience (number of cases). A *P* value < 0.05 was considered to indicate a statistically significant difference.

## 4. Results

Table 2 shows the number of years of ERCP experience and the number of cases wherein ES was performed by the participating endoscopists.

In terms of ERCP experience, 2 endoscopists had no experience, 11 had less than 5 years of experience, 3 had 5–9 years of experience, and 6 had 10 years or more of experience.

Regarding the number of cases wherein ES was performed, 3 beginners had less than 10 cases of experience (of whom 1 had no experience in ES), 12 intermediate-level endoscopists had 10 to 100 cases of experience, and 7 experts had more than 100 cases of experience. A total of 34 ES attempts were made by the 22 endoscopists (mean: 1.5 attempts). Out of these attempts, 33 attempts (97%) resulted in successful cutting. One unsuccessful attempt was made by an endoscopist who had an intermediate level of ES experience and who was unable to manipulate the endoscope to the appropriate direction and thus made an unsuccessful cutting. Six endoscopists attempted NKP 7 times and they succeeded at cutting in the appropriate direction in all attempts. All participants provided their responses to the questionnaire survey and returned the answered questionnaires.

For the feedback on the endoscope manipulation on the dry model compared with the performance of the procedures in actual clinical practice, the overall median score was 6 (range: 3–9). For the question as to whether the dry model provided the realism of cutting as in the actual ES performed in clinical practice, the overall median score was 7 (range: 2–9). According to ES experience, the scores were 7.5 for the beginners, 7 for the intermediate-level endoscopists, and 7 for the experts, showing no statistically significant difference.

For the feedback on NKP performed on the dry model compared with the procedure performed in actual clinical practice, the score was 8 (range: 8–10). For the question as to whether the participants considered the dry model to be useful in improving their skills to perform the ES and NKP techniques, the overall score was 8, that is, 10 for the beginners, 8 for the intermediate-level endoscopists, and 7 for the experts. Although the scores were higher with the ones who have less experience, the differences were not significantly different.

The dry model was considered by the endoscopists at any level of experience to improve their skills. For the question as to whether the participants consider the dry model to be useful for improving their skills in learning how to perform the ES technique, the overall score was 10 (range: 7–10), showing a high evaluation rating (Table 3).

## 5. Discussion

Although ES is frequently performed for biliary disorders, it is also considered to carry a high risk of complications, such as bleeding and perforation. Even though improvement in the papillotome and electrosurgical generators has made the procedure relatively easy, it may still be one of the most challenging procedures for beginners. On the other hand, NKP has a high rate of difficulty, a complications rate ranging from 8.2% to 18.4% and is considered to be a risk factor for post-ERCP pancreatitis [14–17]. At present, beginners acquire skills in performing the ES technique by accumulating experience in actual clinical practice. When trainers instruct beginners, the important points to be covered include how to control the directions of cutting and how long the foot-operated switch of the electrosurgical generators should be stepped on. These points often involve sensory experience and are occasionally difficult to explain with words. Thus, a training model that is easy to use is needed. This is where training by cutting of a realistic dry model may be extremely useful.

The score for the realism of the dry model for ES was 7, indicating that the cutting of the dry model is not completely the same as that of a living human body. The difference may be accounted for by 2 reasons: (1) an uncured ham was used as the simulated papilla and (2) it is difficult to completely reproduce the actual feelings of manipulation of an endoscope in the living body with the duodenum model used in this study. In fact, the score for the manipulation of endoscope operability was slightly low at 6. These factors may have kept the scores for feelings of cutting by ES from exceeding 7. However, even with various models that have been reported, it is difficult to faithfully reproduce the actual feelings of endoscope operation. In the future, the development of gastroduodenal models that closely resemble the living body may be necessary. However, the obtained score for the realism was acceptable and this dry model has proven to be very useful for training simulation to improve ES skills.

There were no significant differences in the results of the questionnaire survey after the hands-on training between the beginner and intermediate groups and the beginner and expert groups. Although some would argue that having an experience of more than 100 cases of ES would make one an expert in this technique, the exact definition of an expert level in terms of ES experience remains unclear. In the present study, we classified the ES expertise level into 3 categories according to the number of ES cases experienced: beginner (<10 cases), intermediate (10–100 cases), and expert (>101 cases). In fact, 6 of the 7 expert endoscopists have more than 10 years of ERCP experience and may have more than 300 cases of ES experience.

For training in the actual cutting, a model on which cutting is actually performed as in this study may be superior to computer simulation in achieving realism. In fact, cutting of organic substances by ES has been reported to be more realistic. Frimberger et al. reported an ES training model using an organic substance with the X-Vision ERCP Training System [18]. Although they have not provided details on what kind of organic substance they used, their model has been assessed to be a satisfactory training model. Matthes and Cohen reported that the Neo-papilla using a chicken heart is useful for training in various ERCP-related procedures, including ES [19]. This model can simulate cannulation of the bile or pancreatic duct using the porcine splenic/iliac artery. Bleeding is reproduced by the injection of red juice to the blood vessels. These features indicate that the dry model is well designed. However, its preparation time of 75 minutes is rather long. Itoi et al. [20] reported a model using a porcine stomach wherein sodium hyaluronate (Mucoup) is locally injected to the porcine gastric mucosa to make it swell and then ES is performed. This model requires the preparation of a porcine stomach. The characteristic feature of this model is the ability to perform cutting with simulated cannulation using a papillotome. Although this is an excellent model, the procedure cannot be performed under the guidance of a guidewire. Thus, each model has its own distinctive features. We believe that the use of simulated papillae made of organic substances for ES training is very useful and realistic. For training endoscopists, having several training models is greatly beneficial. This allows the use of different models depending on the extent of the hands-on training, the number of participants, and the levels of their skills in performing the techniques.

The costs associated with the preparation and use of the training models are also an important issue. The duodenum simulator used in this study is somewhat expensive ($915), but it can be used many times. An uncured ham costs only $0.10 a piece. In the present study, the total cost for the uncured ham used in 34 ES sessions and 7 NKP sessions for the hands-on training was only $4.10.

The limitations of this model include the absence of actual bleeding and perforation. These limitations make it difficult to determine whether the operator is actually cutting in the correct direction. It is therefore necessary to confirm that the cutting is applied in the appropriate direction and appropriate instructions from the supervisor may often be needed. The lack of peristalsis may be another drawback of this dry model. Intestinal peristalsis is one of the factors that make safe ES and NKP difficult. This factor should be considered for any kinds of dry and animal models. This research is also a pilot study, and its assessment is based on the results of a questionnaire survey. Thus, a definitive assessment as to whether this dry model actually contributed to the improvement of skills in the performance of the ES and NKP techniques could not be made. However, based on the feedback that we received from all the endoscopists, they found the dry model to be very useful for the acquisition and improvement of their skills in performing the ES and NKP techniques.

In conclusion, the dry model using an uncured ham for ES and NKP training provides a condition that is closely similar to actual clinical practice and it was found to be useful as a training model. Further study is required to definitively evaluate and measure how this dry model contributes to the acquisition of ES and NKP skills for ERCP endoscopists.

## Figures and Tables

**Table 1:** Questionnaire regarding the dry model.

Please rate the dry model training you are assigned to using the indicated scales

**Q1. How did you feel about the realism of scope manipulation? **
(Please circle a number, where 1 = not realistic; 10 = realistic):
1 2 3 4 5 6 7 8 9 10

**Q2. How did you feel about the cutting sensation of ES?**
(Please circle a number, where 1 = not realistic; 10 = realistic):
1 2 3 4 5 6 7 8 9 10

**Q3. How confident are you that this dry model training would be successful in improving your ES skills?**
(Please circle a number, where 1 = not confident; 10 = confident):
1 2 3 4 5 6 7 8 9 10

**Q4. How confident are you in recommending this dry model to your colleagues who are performing or learning to perform ES?**
(Please circle a number, where 1 = not confident; 10 = confident):
1 2 3 4 5 6 7 8 9 10

**Q5. How did you feel about the cutting sensation of NKP?**
(Please circle a number, where 1 = not realistic; 1 = realistic):
1 2 3 4 5 6 7 8 9 10

**Q6. How confident are you that this dry model training would be successful in improving your NKP skills?**
(Please circle a number, where 1 = not confident; 10 = confident):
1 2 3 4 5 6 7 8 9 10

**Q7. How confident are you in recommending this dry model to your colleagues who are performing or learning to perform NKP?**
(Please circle a number, where 1 = not confident; 10 = confident):
1 2 3 4 5 6 7 8 9 10

ES: endoscopic sphincterotomy; NKP: needle knife precut sphincterotomy.

**Table 2:** ERCP and ES experience of the participants of the hands-on training.

ERCP experience (years)	Number of participants
0	2
<5	11
5–9	3
>10	6
ES experience (number of cases)	
Beginners, <10	3
Intermediate, 10–100	12
Expert, >101	7

**Table 3:** Results of a questionnaire survey after hands-on training.

	Q1. Realism, scope manipulation	Q2. Realism, ES	Q3 Possibility-improving their own ES skill	Q4. Recommend colleagues, ES	Q5. ^†^Realism, NKP	Q6. ^†^Possibility-improving their own NKP skills	Q7. ^†^Recommend colleagues, NKP
All participants	6 (3–9)	7 (2–9)	8 (0–10)	10 (7–10)	8 (8–10)	10 (8–10)	10 (8–10)
ES experience^§^							
Beginners, <10 cases	8 (7–9)	7.5 (6–9)	10 (9-10)	10 (10)	n.a.	n.a.	n.a.
Intermediate, 10–100 cases	5 (3–8)	7 (4–8)	8 (5–10)	10 (7–10)	n.a.	10 (8–10)	10 (8–10)
Expert, >100 cases	6 (4–7)	7 (2–9)	7 (0–8)	10 (8–10)	8 (8–10)	10 (10)	10 (10)

Data presented as median, range.

∗See Table 1 for details of each question.

^†^Only 6 participants performed NKP procedures.

^§^Q1 to Q4: there were no significant differences in the scores between beginner versus intermediate and beginner versus expert.

Q6, Q7: there were no significant differences in the scores between beginner versus intermediate and beginner versus expert.
